# Comparison between Immunization Routes of Live Attenuated *Salmonella* Typhimurium Strains Expressing BCSP31, Omp3b, and SOD of *Brucella abortus* in Murine Model

**DOI:** 10.3389/fmicb.2016.00550

**Published:** 2016-04-20

**Authors:** Won K. Kim, Ja Y. Moon, Suk Kim, Jin Hur

**Affiliations:** ^1^Veterinary Public Health, College of Veterinary Medicine, Chonbuk National UniversityIksan, South Korea; ^2^College of Veterinary Medicine, Gyeongsang National UniversityJinju, South Korea

**Keywords:** *Brucella abortus*, *Salmonella* Typhimurium delivery system, brucellosis, vaccine

## Abstract

Live, attenuated *Salmonella* Typhimurium vaccine candidate expressing BCSP31, Omp3b, and SOD proteins of *Brucella abortus* was constructed. Thirty BALB/c mice were divided equally into three groups, Group A, were intraperitoneally (IP) inoculated with 100 μl of approximately 1.2 × 10^6^ colony-forming units (CFUs)/ml of the *Salmonella* containing vector only in 100 μl as a control. And groups B and C mice were orally and IP immunized with approximately 1.2 × 10^9^ CFU/ml of the mixture of three delivery strains in 10 μl and IP immunized with approximately 1.2 × 10^6^ CFU/ml of the mixture in 100 μl, respectively. The serum IgG, TNF-α and IFN-γ concentrations in groups B (except Omp3b) and C were significantly higher than those in group A. Following challenge with *B. abortus* strain 544; challenge strain was detected <10^3^ CFU from the spleen of all mice of group C. These results suggest that IP immunization with the mixture of the vaccine candidate can induce immune responses, and can effectively protect mice against brucellosis.

## Introduction

Brucellosis is a zoonotic disease cause infections approximately 500,000 people annually worldwide (Corbel, 1997; Pappas et al., 2005, 2006; Akhvlediani et al., 2010). The disease remains endemic in many regions of the world including Latin America, Middle East, Africa, Asia, and the Mediterranean basin (Pappas et al., 2006). *Brucella* is predominantly transmitted to humans via direct contact with fluid discharges from infected animals, but in endemic regions people usually get infected through the consumption of unpasteurized dairy products mainly goat’s milk and fresh soft cheese made out of unpasteurized milk (Avila-Calderón et al., 2013).

Brucellosis by *Brucella* species is facultative intracellular bacteria. *Brucella* infects domestic animals, causing abortion and infertility (Godfroid et al., 2005; Avila-Calderón et al., 2013), however; it can also infect humans producing undulant fever, endocarditis, arthritis and osteomyelitis (Pappas et al., 2005). Both humoral and cell mediated immunities are necessary to change the course of infection of *Brucella*, but cell mediated response is crucial for clearance of *Brucella* from host (Schurig et al., 2002). Th1 type of immune response mediated by IFN-γ helps in clearance of *Brucella* infection (Zhan et al., 1993). Therefore, live and attenuated strains such as *Brucella abortus* strain 19, *B. abortus* RB51, *Brucella melitensis* Rev. 1 and *Brucella suis* strain 2 have been used to control brucellosis in domestic animals (Cheville et al., 1993; Deqiu et al., 2002; Blaco, 2010; Avila-Calderón et al., 2013; Olsen, 2013). These live and attenuated strains may contain a risk because of its potential possibility to revert virulence bacteria (Goel and Bhatnagar, 2012; Avila-Calderón et al., 2013). In addition, these strains can confuse with the diagnosis of brucellosis in serum (Olsen et al., 2009; Avila-Calderón et al., 2013; Olsen, 2013). Because of these limitations, there is necessary for the development of better vaccines which are safer to use.

*Brucella* can cause host infections via mucosa and then the gastrointestinal tract has been known to be one of the major entrances of infection route of brucellosis (Gorvel et al., 2009). Therefore, mucosal immunity is needed to prevent this enteric infection. Oral vaccination can be successful in inducing levels of protective secretory IgA on the intestinal surface (Melkebeek et al., 2013), however, gastric digestion of vaccines before induction of the immune system is a big barrier in the development of oral vaccines (Melkebeek et al., 2013). Live, attenuated *Salmonella* strains have been proposed as an adequate vector to deliver orally heterologous proteins and its use can induces protective mucosal immune responses (Branger et al., 2009; Pathangey et al., 2009; Causey et al., 2010). The essential bacterial gene [such as aspartate β-semialdehyde dehydrogenase (*asd*)]-based balanced-lethal host-vector system has been used to continue plasmids co-expressing inserted antigens in *Salmonella Δasd* mutants and to escape the use of antimicrobials as marker (Cloeckaert et al., 2002; Hur and Lee, 2011b; Olsen, 2013).

Recombinant proteins are a valuable choice for vaccine and can induce antigen specific immune response (Zhao et al., 2009; Goel and Bhatnagar, 2012). An extensively purified protein from Strain 19, the cell surface 31-kilodalton (kDa) protein (or BCSP31), can serve as a protective subunit vaccine in rodents (Tabatabai and Deyoe, 1983; Tabatabai et al., 1989). It has reported that outer membrane proteins (Omps) can be protective antigens of the *Brucella* species (Verstreate et al., 1982). Omp3b, also known as Omp22, belongs to group 3 of the *Brucella* Omps (Vizcaino et al., 2004; Garcia-Yoldi et al., 2005), a highly conserved family that includes the most immunogenic *Brucella* proteins (Cloeckaert et al., 2002). The *sodC* gene encodes a Cu/Zn superoxide dismutase (SOD; Bricker et al., 1990; Sriranganathan et al., 1991). The SOD could function as virulence factor, since it clears harmful oxy-radicals following phagocytosis by macrophages (Tabatabai and Pugh, 1994).

The objective of this study was to evaluate immune responses against *B. abortus* BCSC31, Omp3b and SOD antigens expressing by live attenuated *Salmonella* in a murine model. After *Salmonella* delivery strains with BCSP31, Omp3b, and SOD antigens of *B. abortus* was constructed, mice were immunized by various inoculation routes with the delivery strains. Immune responses induced via intraperitoneal inoculation with the delivery strains were examined in mice. In addition, we evaluated the efficacy of the delivery strains for protection against experimental brucellosis in mice.

## Materials and Methods

### Bacterial Strains and Plasmids

The bacterial strains and plasmids used in present study are listed in **Table 1**. Wild-type *B. abortus* biotype 1 isolated from cattle was used to amplify gene encoding BCSP31, Omp3b, and SOD antigens (**Table 1**). *B. abortus* strain 544 (strain 544) was used as the virulent challenge strain (Lee et al., 2014). The *B. abortus* biotype 1 isolate was kindly supplied by the National Veterinary Research and Quarantine Service, Korea. The attenuated *Salmonella* Typhimurium (*Δlon ΔcpxR Δasd*) mutant strain, JOL912 (Hur and Lee, 2011a) was used as a host for delivery of individual antigens. The pMMP65 plasmid was used as a vector for the expression/secretion of heterologous antigens in the delivery host (Hur and Lee, 2011a). *B. abortus* isolate and strain 544 were grown in Brucella agar (Becton, Dickinson and Company, Sparks, MD, USA). JOL912 was cultured according to the method in previous studies (Kang et al., 2002; Hur and Lee, 2011a,b).

**Table 1:** Bacterial strains and plasmids used in this study.

Strain/plasmid	Description	Source
**Strains**
*S*. Typhimurium
JOL912	*S*. Typhimurium *ΔlonΔcpxRΔasd*	Hur and Lee, 2011a
HJL229	JOL912 with pMMP65	This study
HJL228	JOL912 with pMMP65-BCSP31	This study
HJL219	JOL912 with pMMP65-Omp3b	This study
HJL213	JOL912 with pMMP65-SOD	This study
*B. abortus*
HJL200	*B. abortus* biotype 1 isolate from caw in Korea	Lab stock
HJL254	*B. abortus* strain 544 (ATCC23448)	Lee et al., 2014
**Plasmids**
pET28a	IPTG-inducible expression vector; Km^r^	Novagen
pET32a	IPTG-inducible expression vector; Amp^r^	Novagen
pMMP65	Asd^+^, pBR *ori*, β-lactamase signal sequence-based periplasmic secretion plasmid, 6x His tag	Hur and Lee, 2011a

### Cloning for Recombinant BCSP31, Omp3b, and SOD Proteins

BCSP31, Omp3b, and SOD proteins were prepared from HJL206, HJL204, and HJL208, respectively (**Table 1**), and were used as coating antigens in enzyme-linked immunosorbent assay (ELISA). In addition, these antigens were used as splenocyte stimulating antigens for cytokines concentration measurement. Briefly, genes for BCSP31, Omp3b, and SOD proteins were amplified by polymerase chain reaction (PCR) using specific primer pairs described in **Table 2**. The amplified PCR fragments for each gene were digested with restriction enzymes. Subsequently, the digested fragments were inserted into commercial expression vectors such as pET28a or pET32a. And then these plasmids were transformed into *Escherichia coli* BL21 in order to create HJL206, HJL204, and HJL208 strains. The recombinant BCSP31, Omp3b, and SOD proteins were prepared using an affinity purification process with nickel-nitrilotriacetic acid-agarose (Qiagen, Valencia, CA, USA) from HJL206, HJL204, and HJL208 respectively. Sodium dodecyl sulfate-polyacrylamide gel electrophoresis (SDS-PAGE) was used for the confirmation of the integrity of purified antigens. All purified antigens were stored at -70°C until use.

**Table 2:** Polymerase chain reaction (PCR) primers used in this study and their product sizes.

Primer		Sequence	Size (bp)	Enzyme site	Reference
BCSP31	BCSP31-F	CCGCGAATTCCAGGCCCCGACATTTTTCCG	902	EcoRI	This study
	BCSP31-R	CCGCAAGCTTGGATTATTTCAGCACGCCCGC		HindIII	
Omp3b	Omp3b-F	CCGCGAATTCGCCGACATGATGGGAGGGAC	563	EcoRI	This study
	Omp3b-R	CCGCAAGCTTACTAGAATTTGTAGTTCAGGCC		HindIII	
SOD	SOD-F	CCGCGAATTCAAGTCCTTATTTATTGCATCG	519	EcoRI	This study
	SOD-R	CCGCAAGCTTTTATTCGATCACGCCGCAGG		HindIII	

*Underlines indicate the sites of restriction enzymes.*

### Preparation of *Salmonella* Delivery Strains

The delivery strains were prepared as previously described with a slight modification (Hur and Lee, 2011a). The genes for BCSP31, Omp3b, and SOD proteins were prepared by digestion with restriction enzymes from HJL206, HJL204, and HJL208 strains, respectively. And then each gene was inserted in pMMP65 for the construction of vaccine strains. Subsequently, these plasmids were electroporated into JOL912 to create HJL228 for BCSP31, HJL219 for Omp3b and HJL213 for SOD.

### Western Blot Analysis

Western blot analysis was carried out to check the secretions of the individual BCSP31, Omp3b, and SOD proteins from HJL228, HJL219, and HJL213, respectively, using the modified method mentioned in previous study (Hur and Lee, 2011a). Briefly, the proteins in supernatant of culture were separated by 12% SDS-PAGE gel and transferred onto polyvinylidene fluoride membranes (Millipore, Billerica, MA, USA). The individual proteins were reacted with anti-His antibodies (Invitrogen, Grand Island, NY, USA) and horseradish peroxidase-conjugated rabbit anti-mouse IgG antibodies (Southern Biotech., Birmingham, AL, USA). Immunoreactive bands were detected by addition of the AmershamTM ECL Prime Western Blotting Detection Reagent (GE Healthcare, Little Chalfont, Buckinghamshire, UK) and the CheBi illumination system (Neo science, Suwon, Gyeonggi, South Korea).

### Immunization of Mice and Sample Collection

Thirty 5-weeks-old female BALB/c mice were equally distributed into three groups (*n* = 10 mice per group). All mice were immunized at 6-weeks-old. Group A mice were intraperitoneally (IP) inoculated with approximately 1.2 × 10^6^ colony-forming units (CFUs)/ml of the *Salmonella* containing vector only (JHL229) in 100 μl as a control. Group B mice were orally immunized with approximately 1.2 × 10^9^ CFU/ml of the mixture of the three delivery strains in 10 μl. And group C mice were IP immunized with approximately 1.2 × 10^6^ CFU/ml of the mixture of the three delivery strains in 100 μl. Blood samples were collected before immunization [0 week post-immunization (WPI)] and again at the third WPI for the evaluation of serum IgG. All serum samples were stored at -20°C until use. The animal experiments mentioned in this study were performed under ethics approval (CBU 2015-052) from the Chonbuk National University Animal Ethics Committee in accordance with the guidelines of the Korean Council on Animal Care.

### Immune Response Measurement by Enzyme-Linked Immunosorbent Assay (ELISA)

A standard ELISA was carried out to evaluate the immune response against BCSP31, Omp3b, and SOD antigens in serum samples of mice according to the modified method of the previous study (Hur and Lee, 2011b). Results of the ELISA are expressed as the mean optical density (OD) ± standard deviation.

### Cytokine Quantitation of Splenocytes

At 3 WPI, five mice from each group were sacrificed and spleens were aseptically removed. Splenocytes were prepared according to the method described in previous study (Adone et al., 2012). Splenocytes were distributed in 24 well tissue culture plates with 2 × 10^6^ cells per well (Velikovsky et al., 2003; Cloeckaert et al., 2004). Splenocytes were stimulated *in vitro* with each BCSP31, Omp3b, and SODC antigen (4 μg/well), Concavalin A (0.5 μg/well) as positive control or media as unstimulated control, and incubated at 37°C, 5% CO_2_ and 95% humidity (Adone et al., 2012). The supernatants of reaction were collected after 72 h of re-stimulation and used for cytokine measurement (Adone et al., 2012).

### Cytokines Measurement by ELISA

The concentration of cytokines such as IFN-γ and TNF-α in the supernatants was evaluated by the mouse cytokine ELISA Ready-SET-GO reagent set according to the instructions of manufacturer (eBioscience, Inc., San Diego, CA, USA).

### Challenge Experiments

For challenge experiments, the challenge strain, strain 544 was prepared. Briefly, the strain was cultured in Brucella broth at 37°C for 24 h. and resuspended to approximately 1 × 10^5^ CFU/ml. All mice were IP challenged at 3 WPI with 100 μl of the challenge strain. The spleen weights from all mice were measured and were diluted as 1:100 using Brucella broth. A total of 100 μl of the diluted media was spread on blood agar to count the number of viable strain 544 from the spleens at 2 weeks after the challenge. If no colony detected on the blood agar, means the number of viable challenge strains from the spleen of mice is <10^3^ CFU. The vaccine and challenge strains were confirmed by PCR using *B. abortus*-specific primer (GAC GAA CGG AAT TTT TCC AAT CCC), RB51/2308 primer (CCC CGG AAG ATA TGC TTC GAT CC) and IS*711*-specific primer (TGC CGA TCA CTT AAG GGC CTT CAT) in enhanced *Brucella* AMOS PCR primers (Bricker and Halling, 1994, 1995).

### Statistical Analysis

The number of viable strain 544 from the spleens were normalized by log transformation and estimated by one-way analysis of variance, followed by Tukey’s Multiple Comparison Test using GraphPad program (InStat; GraphPad, La Jolla, CA, USA). The antibody and cellular immune responses were compared between the groups by Kruskal–Wallis test and one-way analysis of variance, respectively using SPSS version 16.0 software (SPSS, Chicago, IL, USA).

## Results

### Secretion of Recombinant BCSP31, Omp3b, and SOD Antigens from Vaccine Candidates

**FIGURE 1:**
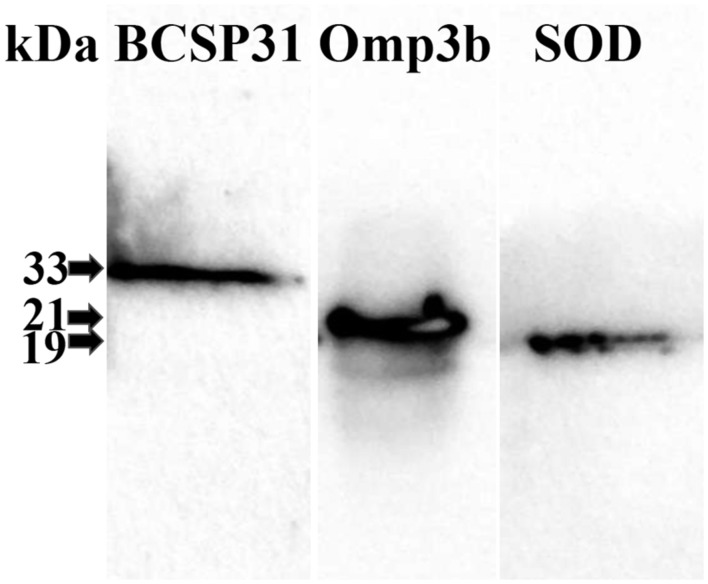
**Identification of recombinant BCSP31, Omp3b, and SOD proteins secreted from attenuated *Salmonella* Typhimurium delivery system using Western blot analysis.** Lanes: BCSP31, recombinant BCSP31 antigen secreted by JHL228; OMP3b, recombinant Omp3b antigen secreted by JHL219; and SOD, recombinant SOD antigen secreted by HJL213.

To express the recombinant BCSP31, Omp3b, and SOD proteins from *Salmonella*, the proteins encoding genes were individually cloned into pMMP65 and were transformed into a *ΔlonΔcpxRΔasd S*. Typhimurium strain. Western blot analysis was carried out to confirm the secretion of BCSP31, Omp3b and SOD proteins from the culture supernatants of the constructs. The expected sizes, 31 kDa for BCSP31, 22 kDa for Omp3b and 19 kDa for SOD, were observed from the precipitated supernatants of culture the individual constructs (**Figure 1**).

### Humoral Immune Responses of the Vaccinated Mice

**FIGURE 2:**
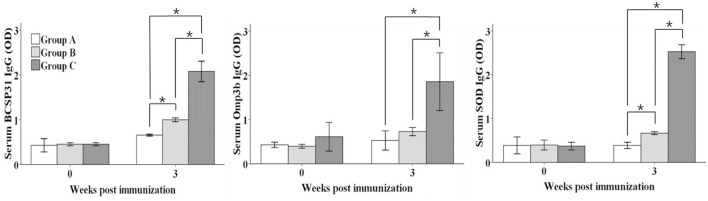
**Serum IgG titers against BCSP31, Omp3b, and SOD antigens.** Group A mice were intraperitoneally (IP) inoculated with approximately 1.2 × 10^6^ CFU/ml of the *Salmonella* containing vector only in 100 μl as a control, group B mice were orally immunized with approximately 1.2 × 10^9^ CFU/ml of the mixture of the three delivery strains in 10 μl and group C mice were IP inoculated approximately 1.2 × 10^6^ CFU/ml of the mixture of the three strains in 100 μl. Data are the mean optical density (OD) of all mice in each group, and error bars show the standard deviations (SD). Asterisks indicate a significant difference between the titers of the immunized group and those of the control group (*P < 0.05).

Antibody responses against each antigen in the serum samples are presented in (**Figure 2**). Serum IgG titers against all antigens in groups B (except Omp3b) and C were significantly increased compared to those of control group (*P* ≤ 0.05). In addition, serum IgG titers against all antigens in group C than group B were significantly higher (*P* ≤ 0.05).

### Cytokine Analysis

**FIGURE 3:**
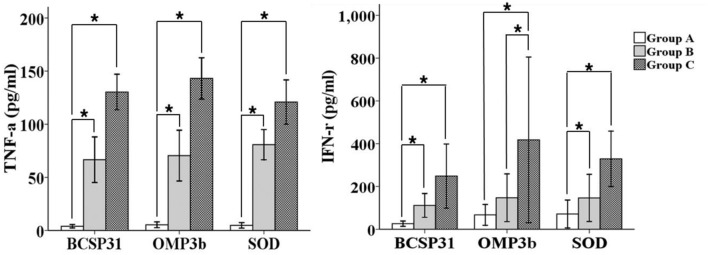
**Cytokine concentrations in the splenocytes at 3 weeks post-immunization.** Groups A–C refer to **Figure 2**. Data are the mean of all mice in each group; error bars show SD. Asterisks indicate a significant difference between the values of each group mice (*P < 0.05).

The TNF-α and IFN-γ concentrations against BCSP31, Omp3b, and SOD antigens in splenocytes re-stimulated with each antigen of mice were measured using ELISA on 3 WPI. The levels of TNF-α and IFN-γ (except Omp3b in group B) were significantly elevated in the groups B and C than group A (*P* < 0.05; **Figure 3**).

### Protection against Challenge

All mice were IP challenged with approximately 1 × 10^4^ CFU of the challenge strain at 3 WPI. The level of protection was evaluated by the number of viable strain 544 from the spleens at 14 days after the challenge. As shown in (**Table 3**), groups B and C mice induced a significantly higher degree of protection than group A. In addition, a significantly higher degree of protection was observed in group C mice than group B mice. Furthermore, among five mice of groups A and B, the challenge strain was isolated from all mice. However, colony was not detected on blood agars spread with broth diluting the spleens of all mice of group C. It means that the number of viable strain 544 from the spleen of group C mice is <10^3^ CFU.

**Table 3:** Bacterial proliferations in spleen of mice challenged with a wild-type *B. abortus* strain 544.

Group	Log10CFU/spleen (mean ± SD)	Significance (P)
A	4.36 ± 0.21	
B	3.48 ± 0.17	<0.05
C	<3.0 ± 0.0	<0.05

*Groups A–C values refer to **Figure 2**.*

## Discussion

The small intestine is a major site of nutrient digestion and absorption (Moreto and Perez-Bosque, 2009). In addition, the intestinal epithelium plays a crucial role in the prevention of pathogenic microorganisms entering the interior space of animals (Moreto and Perez-Bosque, 2009). *B. abortus* can invade hosts through mucosa and is a facultative intracellular bacterium that survives inside phagocytes by escaping the endocytic pathway (Godfroid et al., 2005; Baldwin and Goenka, 2006; Avila-Calderón et al., 2013). The gastrointestinal tract has been known to be one of the major portals of *B. abortus* infection (Gorvel et al., 2009). Therefore, the host resistance to *Brucella* infections depends mainly on mucosal immunity and cell-mediated immunity (Baldwin and Parent, 2002; Paranavitana et al., 2005). Live attenuated *Brucella* vaccines that can stimulate strong mucosal and cell-mediated immune responses are usually very effective against brucellosis (Bricker and Halling, 1994; Murphy et al., 2001; Baldwin and Goenka, 2006). However, live vaccines induce antibodies that confuse the diagnosis of field infection in vaccinated animals (Olsen et al., 2009; Avila-Calderón et al., 2013; Olsen, 2013), which further hinder brucellosis eradication (Velikovsky et al., 2003; Cloeckaert et al., 2004; Izadjoo et al., 2004). In addition, vaccination of pregnant cows with some live vaccine may induce low level abortion or premature birth. Thus it is recommended to be used with caution in pregnant cattle (Olsen et al., 2009; Lin and He, 2012; Avila-Calderón et al., 2013).

In previous studies (Chen and Schifferli, 2003; Hur and Lee, 2011a; Hur et al., 2011, 2014) live attenuatted *Salmonella*, a facultative intracellular bacterium, has been known to induce a strong mucosal immune response as well as cell-mediated immune response. In addition, live attenuated *Salmonella*, as a vector to transfer heterologous proteins, has many benefits such as delivering the protein to the host, and easy preparation and handy inoculation (Chen and Schifferli, 2003; Hur et al., 2011). Especially, oral immunization with live attenuated *Salmonella* vaccine expressing the recombinant rBL protein induced significant protective effect, although this protection was lower than that by the inoculation with the pBL DNA vaccine (Zhao et al., 2009). Major Omps of *Brucella* species have been known to be immunogenic antigens for effective protection against brucellosis (Cloeckaert et al., 2002). In present study, we conducted a new *Brucella* vaccine candidate, attenuated *Salmonella* strain expressing Omps of *Brucella*. We analyzed the immunogenicity and protective efficacy of the vaccine strains against virulent *Brucella* infection in BALB/c mice immunized via various routes with the candidate. BALB/c mouse has been known to be a proven animal model to study the protection of *Brucella* vaccine candidates (Jain et al., 2014).

In the present study, the attenuated *S*. Typhimurium *Δlon ΔcpxR Δasd* strain and the pBP65 plasmid encoding the *asd* gene (Hur et al., 2011) was used as a delivery system to deliver BCSP31, Omp3b and SOD proteins of *B. abortus*. Secretion of each antigen from the vaccine candidate was demonstrated by Western blot analysis. This results indicated that the plasmids carrying the genes for BCSP31, Omp3b, and SOD proteins were stably maintained in the vaccine candidates, and that the recombinant BCSP31, Omp3b, and SOD proteins were effectively expressed and secreted. This combination of three *S.* Typhimurium strains expressing each other protein were determined from preliminary experiments to determine optimal union among various combinations using *S*. Typhimurium strains expressing each protein, such as BCSP31, Omp3b, Omp10, Omp25, SOD. Two different immunization routes of mice, such as oral (group B) and IP (group C), were used to evaluate the immunogenicity and limitation of colonization of *B. abortus* strain 544 in spleen. The mice of groups B (except Omp3b) and C induced significant higher amounts of serum IgG titers to BCSP31, Omp3b, and SOD antigens than mice of control group A. These results show that immunization with the vaccine candidate irrespective of inoculation routes can induce systemic immune response.

*Brucella abortus* is a facultative intracellular bacterium. Therefore, cell-mediated immune response is necessary for clearance of the bacteria (Baldwin and Parent, 2002; Paranavitana et al., 2005). Especially, Th1 type immune response is considered as necessary to protect completely brucellosis (Zhan et al., 1996; Murphy et al., 2001; Baldwin and Goenka, 2006). The role of TNF-α to limit colonization of *B. abortus* in spleen of mice has been known to be critical for activation of macrophages to kill *B. abortus* in the absence of IFN- γ (Zhan et al., 1996; Murphy et al., 2001). IFN- γ plays a critical role in the clearance of *B. abortus* by its ability to activate antibacterial functions of infected macrophages (Paranavitana et al., 2005; Baldwin and Goenka, 2006). We studied the cell mediated immune response generated by various immunization routes with the vaccine candidates expressing BSCP31, Omp3b, and SOD antigens of *B. abortus*. TNF-α and IFN-γ concentrations were evaluated from the supernatants of the re-stimulated splenocytes following restimulation of splenocytes with heat-inactivated *B. abortus* whole antigens of mice immunized with the vaccine candidates. TNF-α and IFN-γ (except Omp3b in group B) concentrations were significantly higher in groups B and C mice than in group A mice. These results indicate that immunization with the conducts irrespective of inoculation routes may induce strong Th1 type immune responses. Furthermore, significantly high levels of IFN-γ as well as TNF-α in splenocyte culture supernatants relative to group B could be observed in group C. It seems logical to conclude that TNF-α and IFN- γ produced by IP immunization with the candidate can have been associated with the increased protection.

The lack of well-established correlation of protection against *Brucella* is a critical difficulty of a novel vaccine candidate (Zhao et al., 2009). In previous study, oral immunization group with live attenuated *Salmonella* vaccine expressing the recombinant fusion protein rBL induced significant protection than control group after oral inoculation with *B. abortus* strain 544 (Zhao et al., 2009). However, the results only showed that the mice immunized with live attenuated *Salmonella* vaccine expressing the recombinant fusion protein could decreased the number of colonization of *Brucella* in spleen (Zhao et al., 2009). Similarly, groups B and C mice relative to group A mice significantly limited colonization of *B. abortus* strain 544 in spleen. These results show that immunization with the candidates irrespective of inoculation routes can protect mice from virulent *B. abortus* strain 544 infection. Furthermore, in this study, group C mice showed the best protection against virulent *B. abortus* strain 544 infection. The reason could be that IP immunization with the candidate coffered large amounts of antigens into antigen-presenting cells, which induced the strongest mucosal and cell-mediated immune responses.

## Conclusion

The results of this study show that immunization with the *Salmonella* delivery system based *Brucella* vaccine induces robust mucosal and cell mediated immune response in mice. Immunization with the vaccine candidate irrespective of inoculation routes confers protection against *B. abortus* infection. Furthermore, in this study, mice IP immunized with the vaccine candidate showed the best protection against *B. abortus* strain 544 infections. On theses bases, we believe that IP immunization with the vaccine candidate can effectively protect brucellosis. In addition, these results imply that combination of live, attenuated *Salmonella* Typhi strains expressing each antigen such as BCSP31, Omp3b, and SOD, may attempt to study as vaccine candidate against human brucellosis, if the attenuated strains should prove safe use in human.

## Author Contributions

WK: performed all the tests in this study and wrote this manuscript; JM: assisted the tests and drew the figures; SK: controlled mice; JH: managed this study and wrote this manuscript with WK.

## Conflict of Interest Statement

The authors declare that the research was conducted in the absence of any commercial or financial relationships that could be construed as a potential conflict of interest.
